# Effects of trimetazidine in combination with bisoprolol in patients with chronic heart failure and concomitant chronic obstructive pulmonary disease: A protocol for systematic review and meta-analysis

**DOI:** 10.1097/MD.0000000000025491

**Published:** 2021-04-16

**Authors:** Xiaoyan Zhang, Shujuan Ma, Baocai Fu

**Affiliations:** a Department of Respiratory Medicine, Changyi People's Hospital; b Department of Respiratory Medicine, Changle People's Hospital; c Department of Critical Care Medicine, Yantaishan Hospital, Shandong, China.

**Keywords:** bisoprolol, chronic heart failure, chronic obstructive pulmonary disease, meta-analysis, protocol, trimetazidine

## Abstract

**Background:**

To the best of our knowledge, there is no study that has conducted a review investigating the clinical efficacy and safety of bisoprolol combined with trimetazidine on chronic heart failure (CHF) patients with chronic obstructive pulmonary disease (COPD). Therefore, in order to provide new evidence-based medical evidence for clinical treatment, we undertook a systematic review and meta-analysis to assess the effectiveness and safety of bisoprolol combined with trimetazidine on CHF patients with COPD.

**Methods:**

Seven electronic databases including Web of Science, Embase, PubMed, Wanfang Data, Scopus, Science Direct, Cochrane Library will be searched in April 2021 by 2 independent reviewers. For search on PubMed, the following search terms will be used: “trimetazidine, bisoprolol, chronic heart failure, chronic obstructive pulmonary disease.” In order to achieve a consistency of extracted items, the data extractors will extract data from a sample of eligible studies. The outcomes include all-cause mortality and hospitalization for cardiac or/and respiratory causes; left ventricular structure and function; and functional scores. Review Manager software (v 5.4; Cochrane Collaboration) will be used for the meta-analysis. Two independent reviewers will assess the risk of bias of the included studies at study level. Any disagreements will be discussed and resolved in discussion with a third reviewer.

**Results:**

The results of our review will be reported strictly following the PRISMA criteria.

**Conclusions:**

The review will add to the existing literature by showing compelling evidence and improved guidance in clinic settings.

**OSF registration number:**

10.17605/OSF.IO/ZWPRB.

## 1 Introduction

Aging is a growing problem - patients with chronic conditions such as chronic obstructive pulmonary disease (COPD), chronic heart failure (CHF), diabetes and hypertension are living longer and in increasing numbers.^[1,2]^ Diseases often coexist in the same patient; COPD and CHF, in particular, result in a significant increase in patients’ quality of life and increased morbidity and mortality due to common risk factors. The prevalence of COPD in patients with CHF ranges from 20% to 32%, while the prevalence of CHF in COPD is more than 20%. This prevalence seems to increase with age.^[3,4]^

Trimetazidine has been evaluated in randomized trials and multiple meta-analyses in patients with CHF. Trimetazidine on top of standard pharmacotherapy has been observed to reduce left ventricular ejection fraction in patients with chronic heart failure, thereby reducing morbidity and mortality.^[5]^ In addition, some studies have shown that trimetazidine may reduce symptoms in the New York Heart Association classification and increase exercise time in CHF.^[5,6]^ Some studies have also shown that trimetazidine can improve cardiopulmonary stress test results and the distance of a six-minute walk.^[7,8]^ Bisoprolol is a highly selective β-adrenergic receptor blocker that has also been shown to be effective in patients with CHF in multiple studies.^[9]^ Although it has been reported that cardio-protective beta blockers do not worsen lung function in CHF patients with COPD, the effect of beta blocker selectivity on long-term prognosis in these patients has not been well evaluated.^[3]^

To the best of our knowledge, there is no study that has conducted a review investigating the clinical efficacy and safety of bisoprolol combined with trimetazidine on CHF patients with COPD. Therefore, in order to provide new evidence-based medical evidence for clinical treatment, we undertook a systematic review and meta-analysis to assess the effectiveness and safety of bisoprolol combined with trimetazidine on CHF patients with COPD.

## 2 Materials and methods

This protocol was written following the Preferred Reporting Items for Systematic Reviews and Meta-Analyses Protocols statement guidelines.

### 2.1 Protocol registration

The prospective registration has been approved by the Open Science Framework (OSF) registries, and the registration number is 10.17605/OSF.IO/ZWPRB. No ethical approval is required in our study because all analyses will be based on aggregate data from previously published studies.

### 2.2 Searching strategy

Seven electronic databases including Web of Science, Embase, PubMed, Wanfang Data, Scopus, Science Direct, Cochrane Library will be searched in April 2021 by 2 independent reviewers. For search on PubMed, the following search terms will be used: “trimetazidine, bisoprolol, chronic heart failure, chronic obstructive pulmonary disease.” To minimize the risk of publication bias, we will conduct a comprehensive search that included strategies to find published and unpublished studies. The reference lists of the included studies will also be checked for additional studies that are not identified with the database search. There is no restriction in the dates of publication or language in the search.

### 2.3 Eligibility criteria

Study included in this review has to meet all of the following inclusion criteria in the PICOS order:

1. population: patients with CHF and COPD;
2. intervention group (group 1): routine treatment with trimetazidine and bisoprolol;
3. comparison group (group 2): routine treatment without trimetazidine and bisoprolol;
4. outcome measures: all-cause mortality and hospitalization for cardiac or/and respiratory causes; left ventricular structure and function; and functional scores;
5. study design: randomized controlled trial or observational study.

Biomechanical studies, in vitro studies, review articles, techniques, case reports, letters to the editor, and editorials are excluded.

### 2.4 Data extraction

In order to achieve a consistency (at least 80%) of extracted items, the data extractors will extract data from a sample of eligible studies. Results of the pilot extraction will be discussed among review authors and extractors. Two independent reviewers will extract data with a predefined extraction template, which includes the following items: study characteristics such as the first author, publication year, study design, follow-up period; patient demographic details such as patients’ number, average age, and gender ratio. The outcomes include all-cause mortality and hospitalization for cardiac or/and respiratory causes; left ventricular structure and function; and functional scores. The original authors will be contacted to request missing data where necessary. Extracted information will be cross-checked by 2 independent reviewers. Any disagreements will be discussed and resolved in discussion with a third reviewer.

### 2.5 Data analysis

Dichotomous data will be analyzed using risk ratio with 95% confidence intervals, whereas continuous variables will be analyzed using weighted mean differences or standardized mean differences. Pooled analyses will be calculated using fixed-effect models, whereas random-effect models will be applied in case of significant heterogeneity across studies. When no events are observed, 0.5 will be added to both arms of the trial. Statistical heterogeneity will be measured using the *I*^2^ statistic. Metaregression analyses will be conducted to estimate the extent to which other covariates may have influenced the treatment effects. Sensitivity analyses will be performed to determine the stability of the overall treatment effects. Additionally, publication bias will be assessed using the Begg adjusted rank correlation test and Egger regression asymmetry test. All *P* values will be 2-tailed, and the statistical significance will be set at 0.05. Review Manager software (v 5.4; Cochrane Collaboration) will be used for the meta-analysis.

### 2.6 Risk of bias

Two independent reviewers will evaluate the risk of bias of the included randomized controlled trials on the basis of the guidelines of the Cochrane Handbook for Systematic Reviews of Interventions 5.1.0 by using Cochrane Collaboration's tool for assessing the risk of bias. The score consists of 7 items, including random sequence generation, allocation concealment, blinding of participants and personnel, blinding of outcome assessment, incomplete outcome data, selective reporting, and other bias. When evaluating the methodological quality of retrospective studies, the Methodological Index for Non-randomized Studies criteria will be used.

## 3 Discussion

COPD is present in approximately one-third of all CHF patients, and is a key cause of underprescription and underdosing of β-blockers, owing largely to concerns about precipitating respiratory deterioration in these patients. To the best of our knowledge, there is no study that has conducted a review investigating the clinical efficacy and safety of bisoprolol combined with trimetazidine on CHF patients with COPD. Therefore, in order to provide new evidence-based medical evidence for clinical treatment, we undertook a systematic review and meta-analysis to assess the effectiveness and safety of bisoprolol combined with trimetazidine on CHF patients with COPD. For this study, our review process will be very rigorous. And this article is a protocol of the systematic review and meta-analysis, which presents the detailed description of review implement. The results of our review will be reported strictly following the PRISMA criteria and the review will add to the existing literature by showing compelling evidence and improved guidance in clinic settings.

## Author contributions

**Conceptualization:** Xiaoyan Zhang, Shujuan Ma.

**Data curation:** Xiaoyan Zhang, Shujuan Ma.

**Formal analysis:** Xiaoyan Zhang, Shujuan Ma.

**Funding acquisition:** Baocai Fu.

**Investigation:** Xiaoyan Zhang, Shujuan Ma.

**Methodology:** Xiaoyan Zhang, Shujuan Ma.

**Project administration:** Baocai Fu.

**Resources:** Baocai Fu.

**Software:** Shujuan Ma.

**Supervision:** Baocai Fu.

**Visualization:** Xiaoyan Zhang, Shujuan Ma.

**Writing – original draft:** Xiaoyan Zhang.

**Writing – review & editing:** Baocai Fu.
